# CoFe-MOF nanoarray as flexible microelectrode for electrochemical detection of catechol in water samples

**DOI:** 10.1016/j.heliyon.2024.e39241

**Published:** 2024-10-11

**Authors:** S. Arivuselvan, Mari Elancheziyan, Raji Atchudan, Deivasigamani Ranjith Kumar, E. Sivasurya, S. Philomina Mary, Pandi Muthirulan, Keehoon Won, Manoj Devaraj

**Affiliations:** a Department of Chemistry, Karpagam Academy of Higher Education, Coimbatore, 641 021, India; b Centre for Material Chemistry, Karpagam Academy of Higher Education, Coimbatore, 641 021, India; c Department of Chemical and Biochemical Engineering, College of Engineering, Dongguk University-Seoul, 30 Pildong-ro 1-gil, Jung-gu, Seoul, 04620, Republic of Korea; d Department of Chemistry, Saveetha School of Engineering, Saveetha Institute of Medical and Technical Sciences, Chennai, 602105, Tamil Nadu, India; e School of Chemical Engineering, Yeungnam University, Gyeongsan, 38541, Republic of Korea; f Centre for Organic and Nanohybrid Electronics, Silesian University of Technology, Konarskiego 22B, 44-100, Gliwice, Poland; g Department of Chemistry, Srimati Indira Gandhi College, (Affiliated to Bharathidasan University), Tiruchirapalli, 620002, India; h Department of Chemistry, Lekshmipuram College of Arts and Science, Neyyoor, 629802, India

**Keywords:** Nanoarray microelectrode, Catechol, Electrochemical sensor, Cyclic voltammetry, Redox mediator, Nonenzymatic sensor, Water samples

## Abstract

A simple, selective, and straightforward enzyme-free electrochemical sensor has been designed and developed using cobalt hexacyanoferrate metal-organic framework (CoFe-MOF) nanoarray. The prepared CoFe-MOF nanoarray has been successfully grown over a carbon cloth (CC) to form CoFe-MOF/CC as a flexible microelectrode for the detection of catechol. The surface of the activated CC was covered uniformly with CoFe-MOF in the form of nanoarray and exhibited double-shelled cubic morphology. The CoFe-MOF/CC nanoarray microelectrode showed a pair of well-defined redox peaks corresponding to the [Fe(CN)_6_]^4-/3-^ redox signal. Interestingly, the fabricated nanoarray microelectrode has displayed superior peak current at lower onset potential with high electrochemical response compared to unmodified potassium hexacyanoferrate (K_3_ [Fe(CN)_6_]) over CC microelectrode and bare activated CC. Further, the developed CoFe-MOF/CC nanoarray microelectrode for the oxidation of catechol was examined with consecutive injections of catechol. A fast and noticeable improvement in oxidation peak current was observed, thus representing the excellent electrocatalytic oxidation of catechol at the modified nanoarray microelectrode. Besides, CoFe-MOF/CC microelectrode exhibits an excellent linear response over a concentration range from 0.005 to 2.8 mM with low detection limit (LOD) and high sensitivity of 0.002 mM (S/N = 3) and 205.99 μA/mM, respectively. Moreover, the prepared nonenzymatic sensor showed outstanding stability, acceptable reproducibility, and repeatability, along with good interference ability. Catechol in spiked water samples was successfully quantified.

## Highlights

• CoFe-MOF was grown successfully on the surface of activated carbon cloth.
• CoFe-MOF as flexible microelectrode was used for detection of catechol.
• The morphology obtained was in the form of double-shelled cubic nanoarrays.
• CoFe-MOF/CC microelectrode exhibited specific selective for catechol detection.

## Graphical abstract

**Figure:**
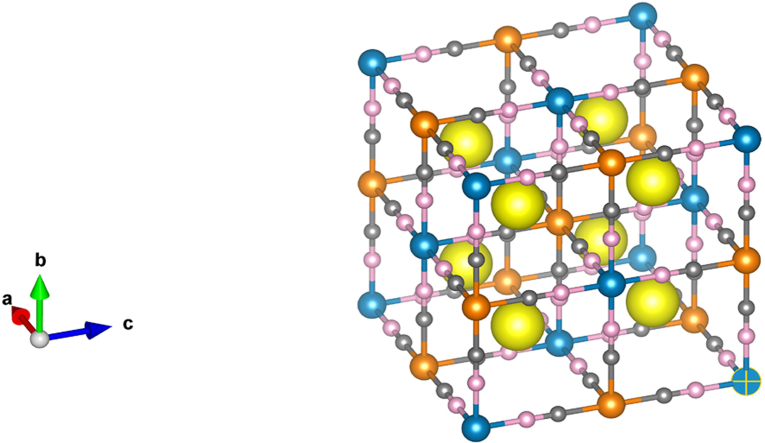

## 1 Introduction

Catechol, also known as 1,2-dihydroxybenzene, is an organic compound that can be found in various natural and anthropogenic sources. The detection of catechol in water samples is essential for several reasons, primarily relating to environmental monitoring and public health [1]. Catechol can enter water bodies through industrial discharges, including those from petrochemical plants, pharmaceutical companies, and other chemical manufacturing processes [2,3]. It is also a breakdown product of some pesticides and other organic substances. Monitoring catechol levels in water helps assess the impact of industrial activity on aquatic environments and assists in the enforcement of environmental regulations [4]. Due to its phenolic structure, it is potentially harmful and has been classified as a pollutant. High concentrations of phenolic compounds like catechol in water can be toxic to aquatic life, affecting the biodiversity and functioning of aquatic ecosystems [5]. For humans, consumption of water contaminated with catechol can be hazardous to health, possibly affecting liver, kidney, and respiratory functions [6,7]. The presence of catechol in water is often an indicator of broader chemical contamination. Since catechol is a degradation product of various synthetic chemicals, its detection can signal the presence of other potentially harmful pollutants. This makes it an essential target in the screening of water quality. Understanding the presence and concentrations of catechol in water bodies can help researchers develop better methods of water treatment and pollution prevention. It also aids in studying the environmental fate of phenolic compounds, which is crucial for developing sustainable industrial practices [8,9]. Up until now, analytical methods such as high-performance liquid chromatography [10], spectrophotometry [11], capillary zone electrophoresis [12], chemiluminescence [13] electrogenerated-chemiluminescence [14], fluorescence [15], and liquid chromatography-fluorescence [16], have been extensively used to detect the presence of catechol in water samples. Although these methods allow for precise quantification, they require highly skilled technicians with high capital costs, and are not fast responsive. Therefore, there is an urgent need to explore other methods that are user-friendly and have low capital costs [17]. Electrochemical detection offers a robust, cost-effective, and highly sensitive method for analysing catechol [18,19]. With the appropriate electrode material and modification, this technique can provide rapid results with high selectivity, making it suitable for a variety of applications from laboratory analyses to field and industrial use. The electrochemical detection of catechol can be performed by either an enzymatic or nonenzymatic pathway by fabricating electroactive materials on conventional electrodes [20]. Even though both techniques have demonstrated their efficacy, the conventional electrodes are non-flexible, sensitive to serve environments, operational and storage stability, and require expensive binders for the fabrication process, which limits their practical usage. On the other hand, flexible microelectrodes exhibit good sensitivity and better operational stability and can be reused several times without losing activity [21,22]. As a result, significant efforts have been directed toward the development of flexible microelectrodes with enhanced electrocatalytic activity by identifying and designing promising electrode materials and/or fabrication processes.

The selection of electrode material plays a vital role in the development of a nonenzymatic sensor, as it significantly affects the sensor's sensitivity, selectivity, catalytic activity, and stability. Therefore, a number of electrode materials/composites are constantly researched, including metal carbides [23], metal oxides [24], metal nanoparticles [25], redox mediators [26], metal complexes [27], carbon nanomaterials [28], polymers [29], metal-organic frameworks (MOF) [30], and others. Among them, MOF-based electrochemical sensors and biosensors are being reported more frequently due to their unique characteristics, such as excellent ionic conductivity, synthetic versatility, good electrochemical stability, low toxicity, and tunable physicochemical properties [31]. The introduction of metals linked with ligands can effectively modify the structure, morphology and increase in active metal sites, and therefore resulted in significant improvement in electrochemical sensing of target analytes. However, the intrinsic low-conductivity and poor stability of MOFs have restricted the sensitive performance. In this regard, significant efforts have been made to design heterometallic MOFs with incorporation of secondary metal species in the framework. Prussian blue analogues are a subclass of MOF that uses metal ions and cyanides to achieve the excellent catalytic activity. Their surface characteristic properties mostly determine the catalytic activity of MOF [32,33]. Therefore, surface alteration is an efficient technique for increasing catalytic activity. Moreover, the introduction of transition metals such as (cobalt, nickel, manganese) to potassium hexacyanoferrate (K_3_ [Fe(CN)_6_]) significantly improves catalytic performance, and this hybrid nanostructure has tremendous promise for achieving high catalytic activity. The introduction of secondary transition metal could provide synergetic effect between heterometallic centres and also create defective sites in structure. It is expected to provide prominent electrochemical features such as increase in signal response, decrease in applied potential and minimized interferences.

Leveraging these advantages, we have prudently designed *in-situ* growth of cobalt hexacyanoferrate metal-organic framework (CoFe-MOF) nanoarray over flexible carbon cloth (CC), which acts as microelectrode and extends the electrochemical application towards the detection of catechol. The introduction of Co into the ferricyanide probe via the cation exchange process can increase the electronic conductivity by decreasing the ohmic resistance and thus enhance the electron transfer ability. The utilization of CC as an electrode substrate enables the uniform growth of CoFe-MOF and direct contact on the electrode surface, which creates an alternative pathway for the rapid designing of flexible microelectrodes. The resultant CoFe-MOF nanoarray exhibited a good electrochemical response towards oxidation of catechol within the linear range from 0.005 to 2.8 mM with a low detection limit (LOD) and high sensitivity of 0.002 mM and 205.99 μA/mM, respectively. Moreover, the prepared nonenzymatic sensor showed good selectivity against various biological and environmental analytes. Therefore, the proposed flexible microelectrode with the loading of electroactive redox species could be an alternative strategy for the detection of a wide range of analytes and biomolecules.

## 2 Experimental section

### 2.1 Materials and reagents

Cobalt chloride hexahydrate, trisodium citrate, K_3_ [Fe(CN)_6_], potassium chloride, hydrochloric acid, catechol, dopamine, ascorbic acid, uric acid, hydroquinone, and glucose were acquired from Sigma-Aldrich, India. CC with a purity of 99.99 % was purchased from the Fuel Cell Store in the United States. All other materials and reagents were of analytical grade and used without further purification. A 0.1 M catechol stock solution was prepared using 0.1 M KCl as a supporting electrolyte. Millipore Milli-Q (MQ) water (resistivity ≥18 MΩ cm) was used for the preparation of electrolytes and synthesis of CoFe-MOF.

### 2.2 Instrumentation

A CHI660E electrochemical workstation (CH Instruments Inc., Texas) with a three-electrode system was utilized for electrochemical measurements under ambient conditions, such as cyclic voltammetry (CV) and amperometric i-t measurements. The bare activated CC, K_3_ [Fe(CN_6_)]/CC, and CoFe-MOF/CC nanoarray microelectrode served as a working electrode. An aqueous Ag/AgCl (3 M KCl) electrode and a platinum coil served as a reference electrode and an auxiliary electrode, respectively. The pH of the supporting electrolyte solution (0.1 M KCl) was monitored by a calibrated pH meter. Field emission scanning electron microscopy (FESEM, Gemini 300 SEM) was utilized for surface roughness and morphology studies of the bare CC and CoFe-MOF/CC nanoarray microelectrode. The elemental mapping of the CoFe-MOF/CC nanoarray microelectrode was performed from energy-dispersive X-ray spectroscopy (EDS). X-ray photoelectron spectroscopy (XPS, PHI Versaprobe III) was carried out to investigate the chemical bond and the electron state at the surface of the CoFe-MOF/CC microelectrodes with an Al Kα monochromatic source. X-ray diffraction (XRD) studies were performed at PANalytical, Netherlands with Cu Kα source (λ = 1.5409 Å, 30 kV, 40 mA).

### 2.3 Fabrication of CoFe-MOF/CC nanoarray microelectrode

**Fig. 1:**
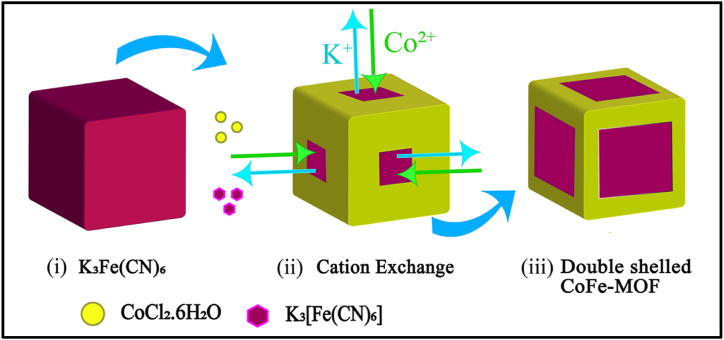
Synthesis of CoFe-MOF/CC double-shelled nanoarray microelectrode.

The CoFe-MOF/CC nanoarray microelectrode was prepared using an in-situ growth method with minor modifications [34]. In a typical synthesis, separately 15 mM of cobalt chloride hexahydrate and 20 mM trisodium citrate were dissolved in 25 mL of MQ water, and the pH of the resulting solution (A) was maintained at 2.0. K_3_ [Fe(CN)_6_] (7.5 mM) was dissolved into 25 mL of MQ water and labelled as sample B. Thereafter, sample B was slowly added into sample A with continuous magnetic stirring for 45 min. The molar ratio of cobalt chloride hexahydrate and K_3_ [Fe(CN_6_)] is maintained at 2:1. After complete addition, the reaction mixture was transferred into a Teflon-lined stainless-steel autoclave. Before the growth of CoFe-MOF nanoarray on CC, the bare CC (geometric surface area of ∼0.48 cm^2^ and a radius of ∼150 μm) was thoroughly rinsed with acetone, ethanol, and water and activated using hydrogen peroxide. Finally, the pre-cleaned CC was immersed in the reaction mixture and maintained at 80 °C for 24 h to obtain CoFe-MOF/CC nanoarray. During the hydrothermal process, the potassium ion was slowly replaced (cation exchange) by a cobalt atom to form the CoFe-MOF nanoarray (Fig. 1). Finally, the prepared CoFe-MOF/CC nanoarray microelectrode (film thickness = ca. 15 μm) was washed with a plenty amount of MQ water to remove the loosely attached molecules before electrochemical investigations. The K_3_ [Fe(CN_6_)]/CC modified electrode were also prepared in similar fabrication procedures without cobalt chloride hexahydrate and trisodium citrate. The detailed experimental procedure are presented in supporting information.

## 3 Results and discussion

### 3.1 Surface morphological investigations

**Fig. 2:**
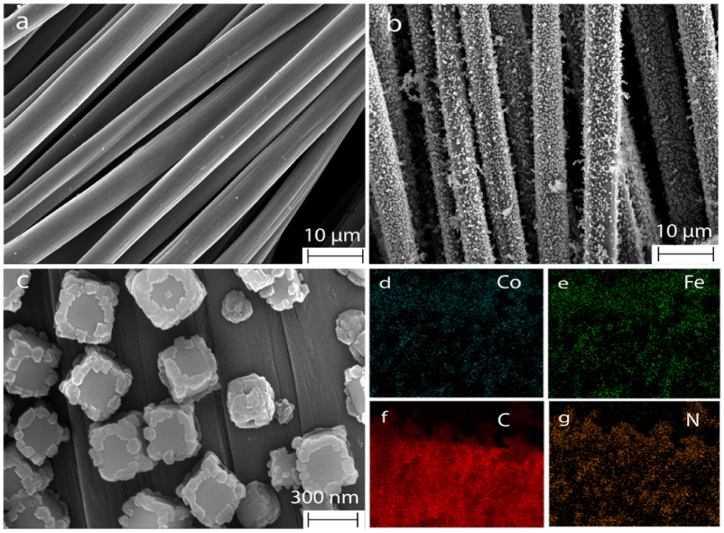
FESEM image of (a) pure activated CC, (b & c) CoFe-MOF/CC nanoarray microelectrode under different magnifications. Elemental mapping of (d) Cobalt, (f) Iron, (f) Carbon, and (g) Nitrogen.

The crystal structure and surface morphology of the activated CC and CoFe-MOF/CC nanoarray were examined by FESEM analysis, and the corresponding elements present in the microelectrode were investigated using mapping analysis. The FESEM images of pure activated CC and CoFe-MOF/CC nanoarray microelectrode (under different magnifications) and the corresponding elemental mapping analysis are given in Fig. 2. It can be clearly seen that the FESEM image of pure activated CC shows transparent surfaces that are uncovered with any of the particles (Fig. 2a) [35]. Further, the reaction with K_3_ [Fe(CN)_6_] and Co precursor resulted in the uniform growth of CoFe-MOF nanoarray on activated CC (Fig. 2b) with well-defined cubic framework (fcc; face center cubic). In closer observation (Fig. 2c), the surface of the ordered cubic framework was covered with a uniform shell layer of Co in the form of a double-shelled structure. The trisodium citrate chelation pathway facilitates the formation mechanism of cobalt hexacyanoferrate. In brief, when cobalt chloride is added in the presence of a chelating agent (trisodium citrate), citrate ions bind with cobalt metal ions effectively to form a cobalt-citrate complex. While adding K_3_ [Fe(CN)_6_], the potassium ions are slowly replaced with cobalt ions via the cation exchange process, thus resulting in the formation of CoFe-MOF after 24 h. Herein, the K_3_ [Fe(CN)_6_] does not undergo a reduction process because of the absence of a reducing agent, and thus, a stable configuration of CoFe-MOF was obtained. After placing the activated CC, the observation of double shell nanoarray reveals the formation of Co on its outer surface as a shell with K_3_ [Fe(CN)_6_] as the core in an ordered manner. Moreover, the CoFe-MOF microcubes were obtained with an average size of 300 nm, which confirms that the CoFe-MOF were successfully grown on the surface of activated CC. The monodispersed cubic framework with a double-shelled layer increases the internal active sites and promotes charge transfer, which is excellent for enhancing the electrocatalytic activity and stability of the catechol sensor. In addition, it can be seen from the outcomes of the elemental mapping images and EDS that cobalt, iron, carbon, and nitrogen were uniformly distributed in CoFe-MOF/CC nanoarray microelectrode (Fig. 2c to g and Fig. S1).

### 3.2 Structural analysis

**Fig. 3:**
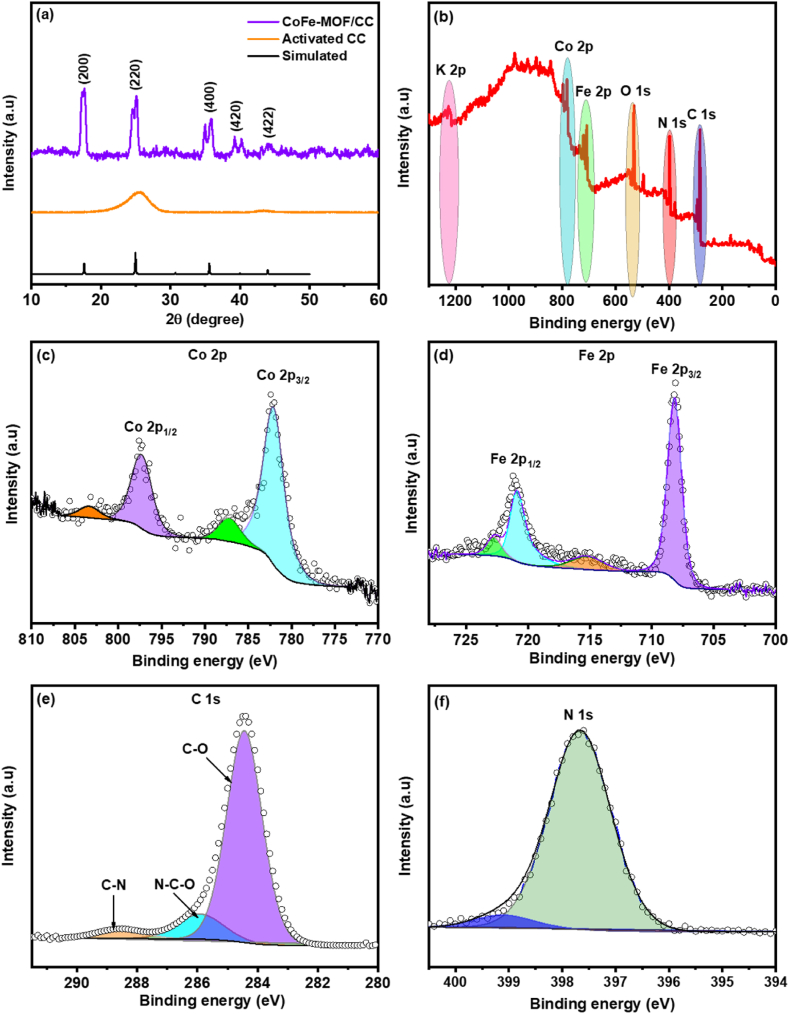
(a) XRD pattern of activated CC and CoFe-MOF/CC nanoarray microelectrode. (b) XPS survey spectrum of CoFe-MOF/CC nanoarray microelectrode. Core level scan of (c) Co 2p, (d) Fe 2p, (e) C 1s, and (f) N 1s.

The structural investigation and phase purity of the activated CC and CoFe-MOF/CC nanoarray microelectrode were performed using the XRD analysis and are displayed in Fig. 3a. For activated CC, the broad peaks located at 26° and 43° corresponding to the (002) and (101) plane indicates the carbon cloth material is composed solely of carbon in the absence of any trace metal impurities [36]. After the CC was modified with CoFe-MOF, the observation of sharp and strong diffraction peaks can be indexed to CoFe-MOF which are well-matched with JCPDS card no. 01-075-0038, with a lattice constant of a = b = c = 1.008 nm (space group: *F-43m*, crystal: *cubic unit*). In addition, no differential peaks were found other than those of CoFe-MOF and the CC, implying the exceptional purity of the CoFe-MOF/CC nanoarray microelectrode. To gain further insight into the surface chemical composition and electronic state of the elements in CoFe-MOF/CC nanoarray microelectrode, XPS analysis was carried out. As shown in Fig. 3b, the survey spectrum confirms the presence of Co and Fe elements along with C 1s, N 1s, O 1s, and K 2p in the CoFe-MOF/CC nanoarray microelectrode. The binding energies were observed at 715 eV, 790 eV, 285 eV, 398 eV, 530 eV, and 1120 eV, which are characteristic of the Fe 2p, Co 2p, C 1s, N 1s, O 1s, and K 2p regions, respectively. The individual elemental peaks along with their deconvoluted peaks originating from the presence of underlying chemical species CoFe-MOF/CC nanoarray microelectrode, are shown in Fig. 3c–f. The deconvoluted XPS spectrum of Co 2p (Fig. 3c) exhibits two characteristic peaks located at 797.7 and 782 eV corresponding to the Co 2p_3/2_ and Co 2p_1/2_, along with the appearance of satellite peaks, respectively, signifying the oxidation state of Co in either Co^2+^ or Co^3+^ [37]. The formation of Co^2+^ in CoFe-MOF further confirms the cation exchange process occurred by K^+^ ion with Co^2+^ ion, thus resulting in the formation of stable CoFe-MOF. Similarly, the Fe 2p spectrum of CoFe-MOF/CC nanoarray microelectrode can be deconvoluted into Fe 2p_3/2_ and Fe 2p_1/2_ regions with the peaks located at 708 and 720.8 eV, along with the satellite peaks at 715 eV and 723 eV which are related to insertion of Fe ions coordinated with N coordinated sites as high spin Fe^2+^ (Fig. 2d) [38]. The C 1s spectrum in Fig. 3e shows the three bands at 285 eV, 287 eV, and 289 eV corresponding to the C–O, N–C–O, and C–N, respectively. Further, the N 1s (Fig. 3f) visualizes the peak at 397.5 eV which corresponds to the existence of nitrogen in cyano ligand, and the observation of smaller band at 399.4 eV corresponds to the presence of ferricyanide unit in CoFe-MOF [39,40]. The presence of mixed valence states among the multi-metals significantly offers high internal surface area, excellent electrical conductivity, and ionic mobility, resulting in the enhancement of the electrocatalytic activity and structural stability of the newly prepared CoFe-MOF/CC nanoarray microelectrode.

### 3.3 Electrochemical characteristic of CoFe-MOF nanoarray microelectrode

**Fig. 4:**
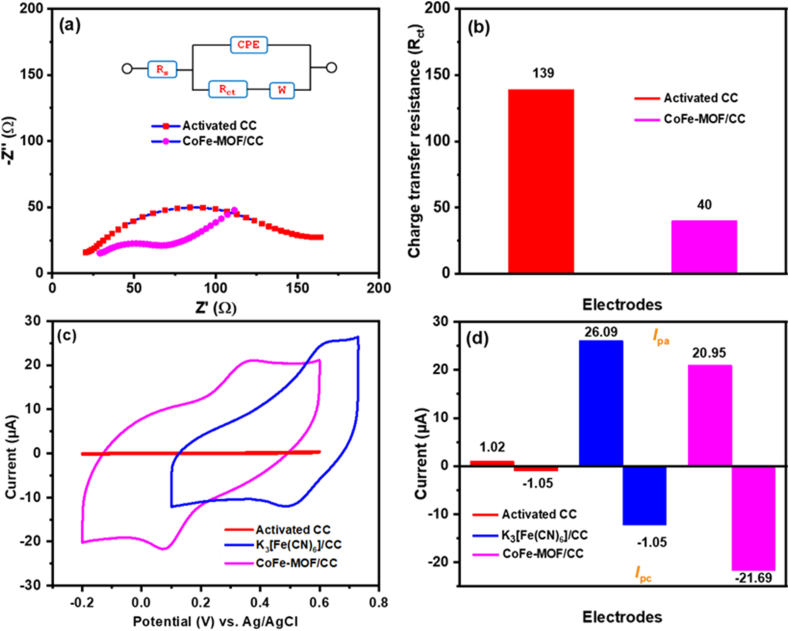
(a) EIS curves of bare activated CC and CoFe-MOF/CC were measured in 0.1 M KCl containing 2.5 mM [Fe(CN)_6_]^3-/4-^. (b) Histogram bar diagram shows *R*_ct_ for the activated CC and CoFe-MOF/CC. (c) CVs cures of activated CC, K_3_ [Fe(CN)_6_]/CC, and CoFe-MOF/CC were recorded in 0.1 M KCl. (d) Histogram bar shows *I*_pa_ and *I*_pc_ responses for activated CC, K_3_ [Fe(CN)_6_]/CC, and CoFe-MOF/CC.

To demonstrate the electrochemical properties of the constructed nonenzymatic sensor, electrochemical impedance spectroscopy (EIS) was performed on modified and unmodified nanoarray microelectrodes to understand the surface alteration through impedance change that occurred during the step-wise nanoarray microelectrode fabrication. Fig. 4a depicts the EIS curves for bare activated CC and CoFe-MOF/CC nanoarray microelectrode, which were recorded over a frequency range of 1 MHz–0.01 MHz and with an amplitude of 10 mV in 0.1 M KCl containing 2.5 mM [Fe(CN)_6_]^3-/4-^ at the formal potential of the redox signal. The impedance spectral data was fitted using the Randles equivalent circuit, which could provide quantitative information on impedance of the faradaic reaction: solution resistance (*R*_s_) in series with the parallel combination of a constant phase element (CPE) and a series of charge transfer resistance (*R*_ct_) and Warburg impedance (W) (inset of Fig. 4a). The EIS curve of activated bare CC shows a well-defined semicircle at a higher frequency region with *R*_ct_ of 139 Ω, which means the inadequate electron transfer process at the activated bare CC [41]. Interestingly, with further modification of Co into K_3_ [Fe(CN)_6_] (CoFe-MOF/CC nanoarray microelectrode), the *R*_ct_ value drastically decreased to 40 Ω, representing the superior electron transfer shuttling ability of the developed CoFe-MOF/CC nanoarray microelectrode. Compared to bare activated CC microelectrode, the CoFe-MOF/CC nanoarray microelectrode offers a large internal active surface area as well as excellent conductivity due to the presence of the mediating nature of Co, thus making it an appropriate platform for the development of the nonenzymatic electrochemical sensor. The *R*_ct_ values for the bare and modified CC electrodes are displayed in Fig. 4b.

The electrochemical redox characteristic behaviour of the bare activated CC, K_3_ [Fe(CN)_6_]/CC, and CoFe-MOF/CC nanoarray microelectrode was investigated using the CV technique, and the results are displayed in Fig. 4c. The CV of these modified and unmodified electrodes was performed in 0.1 M KCl as background electrolytes at a scan rate of 50 mV/s. As anticipated, the activated CC did not find any redox signal in the optimized potential window; this may be a non-redox active signal of the background electrolytic solution. On the other hand, the native K_3_ [Fe(CN)_6_] (K_3_ [Fe(CN)_6_]/CC nanoarray microelectrode) portrayed a set of well-resolved redox signals with the higher potential region, characteristic of [Fe(CN)_6_]^4-/3-^ redox signals with an anodic and cathodic peak potential (*E*_pa_ and *E*_pc_) at 0.619 and 0.492 V, respectively. Interestingly, the newly developed CoFe-MOF/CC nanoarray microelectrode displayed a similar set of well-defined redox signals with the lower potential region when compared to K_3_ [Fe(CN)_6_]/CC nanoarray microelectrode, and the peak potentials located for *E*_pa_ and *E*_pc_ were observed at 0.355 and 0.072 V, respectively. These outcomes confirm that the CoFe-MOF was effectively grown on the activated CC, which coincide well with the XPS results. The distinct performance of CoFe-MOF/CC nanoarray microelectrode is accredited to the integration of Co atom into the sensor design, which boosted their electrochemical properties by improving their internal surface area and electrical conductivity, resulting enhancing their detection limit, sensitivity, and selectivity. Due to these unique advantages, the developed nanoarray microelectrode could be successfully utilized for the nonenzymatic voltammetric determination of catechol. Fig. 4d shows the bar diagram of *I*_pa_ and *I*_pc_ responses of the bare and modified CC electrodes.

The determination of electrochemically active surface area (ECSA) of bare CC, K_3_ [Fe(CN)_6_]/CC, and CoFe-MOF/CC nanoarray microelectrode can be examined by the Randles-Sevcik equation [42]: *I*_pa_ = (2.69 × 10^5^) *n*^3/2^ *AD*^1/2^ *C*υ^1/2^; where *I*_p_ is the peak current (A), *D* is the diffusion coefficient (6.2 × 10^−6^ cm^2^s^−1^ for [Fe(CN)_6_]^3-/4-^), υ is the scan rate (mV/s), *n* is the number of electron involved in the redox process (unity for [Fe(CN)_6_]^3-/4-^), *C* is the bulk concentration of electroactive species, and *A* is the ECSA of the Bare CC and CoFe-MOF/CC nanoarray microelectrode (cm^2^). Therefore, the ECSA could be calculated from the slope of *I*_pa_ vs scan rate. The calculation displays that the ECSA with CoFe-MOF/CC nanoarray microelectrode exhibits a higher ECSA (0.364 cm^2^) value than the activated CC (0.216 cm^2^) and K_3_ [Fe(CN)_6_]/CC nanoarray microelectrode (0.276 cm^2^). The higher ECSA observed for the CoFe-MOF/CC nanoarray microelectrode suggests that CoFe-MOF support effectively promotes the dispersion of CoFe-MOF nanoarray, resulting in an increase in the internal electrochemical active site as well as conductivity of the microelectrode.

**Fig. 5:**
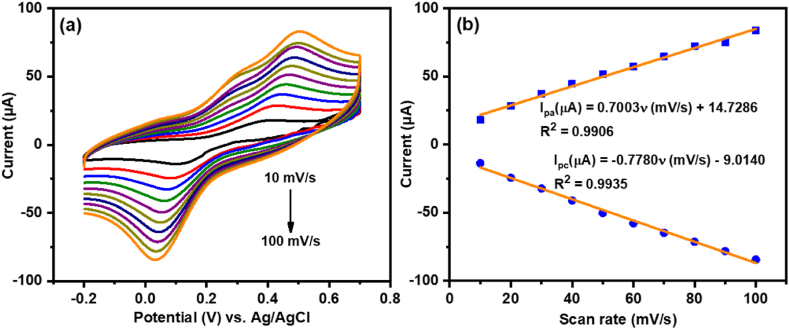
(a) Cyclic voltammograms of CoFe-MOF/CC nanoarray microelectrode at various scan rates from 10 to 100 mV/s in 0.1 M KCl containing 15 μM catechol. (b) Corresponding linear plot of oxidation and reduction peak currents against scan rates.

The voltammetric performance of the developed nonenzymatic sensor was further investigated by CV at different scan rates from 10 to 100 mV/s in 0.1 M KCl electrolyte solution containing 15 μM catechol and the obtained results are displayed in Fig. 5a. As can be seen, the current densities of both *I*_pa_ and *I*_pc_ of CoFe-MOF/CC nanoarray microelectrode were linearly increased with an incremental scan rate ranging from 10 to 100 mV/s. From the calibration plot, the *I*_pa_ and *I*_pc_ peak currents were linearly proportional to the scan rate, which suggests that the redox process of catechol at CoFe-MOF/CC nanoarray microelectrode was the surface-controlled process (Fig. 5b) [43]. Moreover, with the increasing scan rate from 10 to 100 mV/s, the *E*_pa_ was moved slightly in the positive and *E*_pc_ was moved slightly in the negative direction, indicating that the electrochemical redox reaction of catechol at CoFe-MOF/CC nanoarray microelectrode is reversible.

### 3.4 Electrocatalytic oxidation of catechol at CoFe-MOF/CC nanoarray microelectrode

**Fig. 6:**
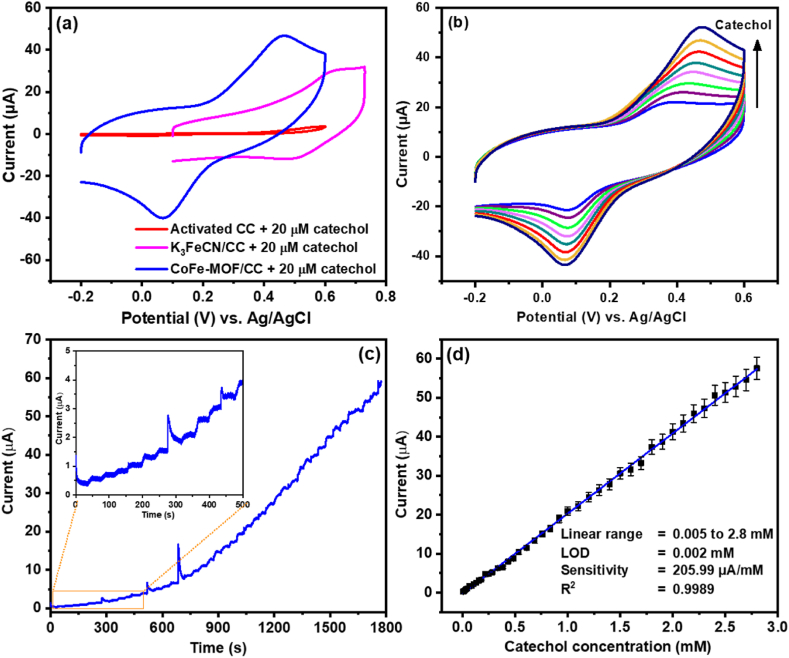
(a) CVs of bare activated CC, K_3_ [Fe(CN)_6_]/CC, and CoFe-MOF/CC nanoarray microelectrode in 0.1 KCl containing 20 μM catechol. (b) CVs of CoFe-MOF/CC nanoarray microelectrode with increasing concentration of catechol. (c) Amperometric i-t response of CoFe-MOF/CC nanoarray microelectrode with increasing concentration of catechol at +0.45 V. (d) Corresponding linear curve (current vs concentration of catechol).

The excellent electrochemical behaviour of the CoFe-MOF/CC nanoarray microelectrode inspired us to examine the electrocatalytic ability of the fabricated nonenzymatic sensor. Accordingly, the electrocatalytic performance of the activated CC, K_3_ [Fe(CN)_6_]/CC, and CoFe-MOF/CC nanoarray microelectrode was studied in 0.1 M KCl at a scan rate of 50 mV/s and the outcomes are depicted in Fig. 6a and b. Upon successive addition of catechol (0–25 μM), the oxidation peak current (*I*_pa_) gradually increased, which denotes that the electrocatalytic oxidation of catechol is favourable at CoFe-MOF/CC nanoarray microelectrodes. During the anodic scan, [Fe(CN)_6_]^4-^ gets oxidized to [Fe(CN)_6_]^3^^-^ by electrochemical oxidation. The resultant, [Fe(CN)_6_]^3-^ electrochemically oxidizes the spiked catechol to *o-quinone* and becomes reduced back [Fe(CN)_6_]^4^^-^ , which can be confirmed by an increase in the oxidation current response as given in Fig. 6b. This electrocatalytic reaction process occurs as long as catechol is present at the modified electrode surface. In addition, to investigate the efficiency of the developed sensor, the electrocatalytic behaviour was compared with activated CC and K_3_ [Fe(CN)_6_]/CC nanoarray microelectrodes under the same experimental conditions (Fig. 6a). The CV responses of activated CC and K_3_ [Fe(CN)_6_]/CC nanoarray microelectrodes showed that there was a feeble increment with higher oxidation compared to CoFe-MOF/CC nanoarray microelectrode. The poor electrocatalytic response at bare-activated CC and K_3_ [Fe(CN)_6_]/CC nanoarray microelectrode are not appropriate for catechol quantification. Moreover, the electrocatalytic response of CoFe-MOF/CC nanoarray microelectrode is ∼10 times higher than that of activated CC microelectrode. These results clearly denote that the enhanced electrocatalytic response is due to the integration of the Co atom into the outer sphere of [Fe(CN)_6_] microelectrode, which displayed a significant current increase for every consecutive injection of catechol.

After a successful examination of the electrocatalytic response to catechol at CoFe-MOF/CC nanoarray microelectrode under static conditions, their efficiency was further investigated under dynamic conditions (amperometric i-t technique). Therefore, amperometry was performed using CoFe-MOF/CC nanoarray microelectrode under optimized experimental parameters with sequential injection of catechol into KCl (0.1 M; 300 rpm) at a working potential of +0.45 V, and the results are portrayed in Fig. 6c. For every injection of catechol, the oxidation current was gradually increased for CoFe-MOF/CC nanoarray microelectrode, which exhibited a linear and stable electrocatalytic response with the steady state i-t of 96 % stabilized within 5 s, representing the fast electrocatalytic activity and quick electron transfer kinetics of anew fabricated sensor. Fig. 6d depicts the plot of oxidation current against the concentration of catechol, in which CoFe-MOF/CC nanoarray microelectrode displayed a broad linear response over the catechol concentration range from 0.005 to 2.8 mM with a low detection limit (LOD) and high sensitivity as 0.002 mM (S/N = 3) and 205.99 μA/mM, respectively. The LOD was calculated using formula 3σ/*m*, where σ represents the standard deviation blank electrochemical measurements (10 measurements), and the sensitivity was calculated using the slope (*m*) obtained from the calibration plot. Moreover, the working concentration range, method, LOD, and real sample of the CoFe-MOF/CC nanoarray microelectrode are comparable to or better than those of the recently published catechol sensors (Table 1). The excellent electrocatalytic performance of the CoFe-MOF/CC nanoarray microelectrode could be the integration of Co into the K_3_ [Fe(CN)_6_] structure, which enriched the electrochemical properties.

**Table 1:** Comparison between the different modified electrodes for the determination of catechol with proposed techniques.

Electrodes	Linear range (μM)	LOD (μM)	Method	Real sample	Ref
NiCoFe-LDH NFs/CC	5–200	0.11	DPV	Tap and Lake	[44]
OV-LDHS/H-MWCNTs/GCE	0.5–150	0.074	DPV	Wastewater	[45]
ZnO/carbon cloth	2–45	0.81	DPV	–	[46]
CoFe_2_Se_4_/PCE-2/GCE	0.5–190	0.15	DPV	Lake	[47]
Co_x_Fe_3_-xO_4_-β-CD/GCE	1–200	0.12	DPV	Tap, River, and Pond	[48]
Meso-Co_3_O_4_	1–500	0.1	DPV	Tap	[49]
Zn/Co-ZiF NPAs/CFC	2–500	0.06	DPV	Lake and River	[50]
AuNPs/Cs@N,S MWCNTs	1–1000	0.2	Amperometry	Tap	[51]
Co_3_O_4_@carbon/GCE	0.6–116.4	0.03	DPV	River	[35]
CoFe-MOF/CC	5–2800	2.0	Amperometry	Tap and Lake	This work

### 3.5 Selectivity, stability, and reproducibility of the CoFe-MOF/CC nanoarray microelectrode

**Fig. 7:**
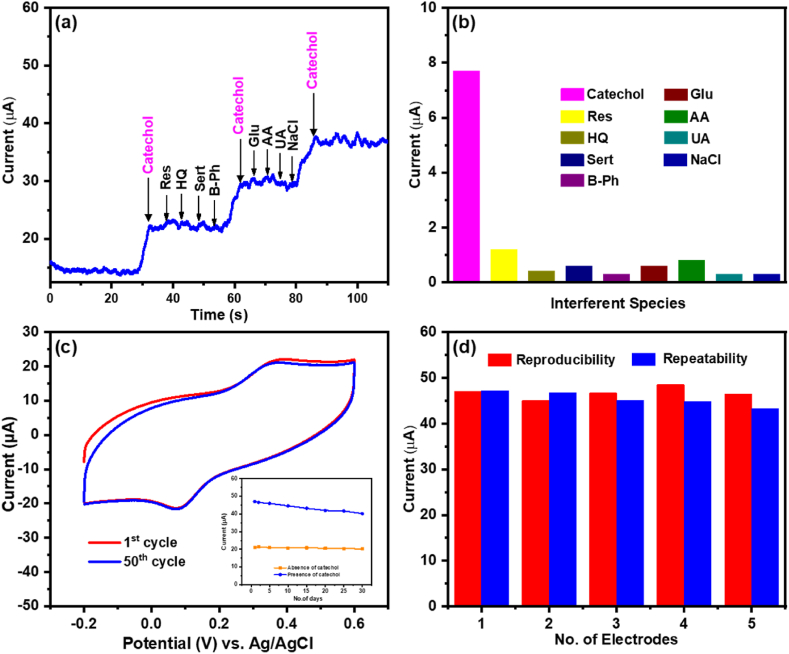
(a) Influence of electroactive interferent species (10-time excess concentration of resorcinol, hydroquinone, sert, B-Ph, glucose, ascorbic acid, uric acid, sodium chloride) on the response of 20 μM catechol. (b) Corresponding columnar diagram of the tested electroactive interferent species compared with catechol. (c) CVs of CoFe-MOF/CC nanoarray microelectrode in 0.1 M KCl for 1st cycle (red curve) and 50th cycle (blue curve) at a scan rate of 50 mV/s. (d) Demonstration of reproducibility and repeatability of the fabricated sensor.

Selectivity is an important phenomenon for any enzyme-free electrochemical sensor to determine its efficacy and practical usage. In particular, the presence of hydroquinone (HQ) and resorcinol (Res) is a cause for concern because they share similar structural properties and chemical activity with catechol, which might readily interfere with the oxidation signal during analysis. Moreover, the selectivity of the proposed nonenzymatic sensor is more significant in the presence of the environmental and biological compounds that are oxidized at potentials to a catechol oxidation potential. Therefore, using the amperometric technique, we tested the selectivity of the sensor in the presence of potentially active environmental and biological compounds, such as resorcinol (Res), hydroquionone (HQ), serotonin (sert), bisphenol (B-Ph), glucose (Glu), ascorbic acid (AA), uric acid (UA), and sodium chloride (NaCl) in a stirring KCl (0.1 M) containing 20 μM catechol (Fig. 7a). It could be seen that the i-t current response of the CoFe-MOF/CC nanoarray microelectrode increased upon injection of only 20 μM catechol and did not increase to other coexisting compounds with ten-fold excess concentrations except Res (∼14 %) and AA (∼10 %). Fig. 7b depicts the corresponding oxidation current response of the tested electroactive interferent species. These results clearly substantiate that the fabricated CoFe-MOF/CC nanoarray microelectrode has displayed excellent selectivity for the nonenzymatic determination of catechol.

The stability of the CoFe-MOF/CC nanoarray microelectrode was investigated by continuous potential cycling (CV technique) in 0.1 M KCl at a scan rate of 50 mV/s, and the results are depicted in Fig. 7c. It was observed that even after 50 consecutive potential cycles, the anodic and cathodic peaks showed no substantial change, indicating the CoFe-MOF/CC nanoarray microelectrode has good structural stability. Additionally, the long-term stability of CoFe-MOF/CC nanoarray microelectrode was examined in the presence and absence of 10 μM catechol for one month (Inset to Fig. 7c). According to this study, at the end of the month, the fabricated CoFe-MOF/CC nanoarray microelectrode retained 97 % and 94 % of its initial response in the absence and presence of catechol, respectively. The exceptional stability of the CoFe-MOF/CC nanoarray microelectrode could be due to the integration of Co into the K_3_ [Fe(CN)_6_] structure (cation exchange), which significantly enhanced the electrochemical performance of the nonenzymatic sensor. Moreover, the reproducibility of the fabricated sensor was examined by preparing five individual CoFe-MOF/CC nanoarray microelectrodes utilizing the same experimental protocol and then by measuring their electrocatalytic oxidation current response with 10 μM catechol (Fig. 7d red colour). The RSD (relative standard deviation) was found to be 2.67 % in the presence catechol, which demonstrates remarkable electrode-to-electrode reproducibility. Further, the repeatability of the fabricated CoFe-MOF/CC nanoarray microelectrode was investigated using CV in the presence of catechol (five times) with the same nanoarray microelectrode (Fig. 7d blue colour). The RSD was found to be 3.58 %, which suggested excellent repeatability of the fabricated nanoarray microelectrode.

### 3.6 Determination of catechol in real samples

The practical feasibility of the CoFe-MOF/CC nanoarray microelectrode was evaluated by to quantifying the catechol in real water samples. The water samples were collected from a tap water at a University lab and lake water in India, and labelled as samples #1 and #2, respectively. This experiment was conducted under the optimal conditions, wherein the water samples were diluted 10-fold using 0.1 M KCl. The standard addition method was used, in which known concentrations of catechol were injected into the water samples. The recoveries were analyzed using an amperometry current response and were found to range from 91.9 to 103.3 % (Table 2). Thus, the newly prepared CoFe-MOF/CC nanoarray microelectrode nonenzymatic sensor shows an outstanding ability to quantify catechol in real samples, making it suitable for use with practical samples.

**Table 2:** Analysis of catechol in water samples.

Sample	Spiked (μM)	Found (μM)	Recovery (%)	RSD^a^ (%)
**Tap water #1**	5.0	4.61	92.2	2.54
	10	9.19	91.9	2.42
	15	15.2	101.3	2.22
**Lake water #2**	5	4.87	97.4	2.84
	10	9.81	98.1	2.64
	15	15.5	103.3	2.32

## 4 Conclusions

In conclusion, a facile and rapid in-situ fabrication of flexible CoFe-MOF microelectrode was developed using an ion exchange reaction between potassium hexacyanoferrate and cobalt chloride. The CoFe-MOF grown on the carbon cloth was obtained in the form of nanoarray, which acted as a redox probe for facilitating the electrochemical oxidation of catechol. The developed bimetallic CoFe-MOF microelectrode exhibited a good electrochemical response towards catechol, which might be due to the following factors: (1) the introduction of cobalt layer as bimetal, which cover Fe-MOF evenly, induces the facile oxidation of catechol at lower potentials with high current density; (2) the integration of secondary metal atoms provides a synergetic effect, which allows improved electronic-coupling interactions between Co and Fe metal ions; (3) the binder-free and free-standing CoFe-MOF flexible microelectrode provides continuous redox channels for facile electron transfer, which accelerates the oxidation of catechol through an electrode-electrolyte interface. The bimetallic CoFe-MOF microelectrode exhibited a good sensitivity of 205.99 μA/mM, a wide linear range of 0.005–2.8 mM and was also selective for catechol against various interference species. Therefore, the present work highlights the essential pathway for designing and incorporating redox active species on the electrode surface, and this provides the development of highly sensitive flexible electrodes at a lower cost.

## CRediT authorship contribution statement

**S. Arivuselvan:** Visualization, Validation, Methodology, Investigation. **Mari Elancheziyan:** Writing – original draft, Visualization, Validation, Investigation. **Raji Atchudan:** Validation. **Deivasigamani Ranjith Kumar:** Validation, Conceptualization. **E. Sivasurya:** Methodology. **S. Philomina Mary:** Validation. **Pandi Muthirulan:** Visualization, Validation. **Keehoon Won:** Writing – review & editing, Conceptualization. **Manoj Devaraj:** Writing – review & editing, Writing – original draft, Supervision, Funding acquisition, Conceptualization.

## Data availability

Data will be made available on request.

## Declaration of competing interest

The authors declare that they have no known competing financial interests or personal relationships that could have appeared to influence the work reported in this paper.
