# Supplementary material for: CoFe-MOF nanoarray as flexible microelectrode for electrochemical detection of catechol in water samples
Source: Heliyon. 2024 Oct 11;10(20):e39241. doi: 10.1016/j.heliyon.2024.e39241 (PMC11620242; doi:10.1016/j.heliyon.2024.e39241)
Supplement: Multimedia component 1 [file mmc1.docx]

**Electronic Supplementary information**

**CoFe-MOF nanoarray as flexible microelectrode for electrochemical detection of catechol in water samples**

S. Arivuselvan^a,b†^, Mari Elancheziyan^c†^, Raji Atchudan^d,e†^, D. Ranjithkumar^f^, E. Sivasurya^a^, S. Philominamary^g^, P. Muthirulan^h^, Keehoon Won^c^, Devaraj Manoj*^a,b^

*^a^ Department of Chemistry, Karpagam Academy of Higher Education, Coimbatore- 641 021, India*

*^b^Centre for Material Chemistry, Karpagam Academy of Higher Education, Coimbatore- 641 021, India*

*^c^Department of Chemical and Biochemical Engineering, College of Engineering, Dongguk University-Seoul, 30 Pildong-ro 1-gil, Jung-gu, Seoul 04620, Republic of Korea. ^d^Department of Chemistry, Saveetha School of Engineering, Saveetha Institute of Medical and Technical Sciences, Chennai 602105, Tamil Nadu, India.*

*^e^School of Chemical Engineering, Yeungnam University, Gyeongsan 38541, Republic of Korea.*

*^f^Centre for Organic and Nanohybrid Electronics, Silesian University of Technology,*

*Konarskiego 22B, 44-100 Gliwice, Poland*

*^g^Department of Chemistry, Srimati Indira Gandhi College, (Affiliated to Bharathidasan University), Tiruchirapalli-621002, India.*

*^h^Department of Chemistry, Lekshmipuram College of Arts and Science, Neyyoor-629802*

*Corresponding Author: Department of Chemistry, Karpagam Academy of Higher Education, Coimbatore 641021, Tamil Nadu, India.

Email: manojdvrj@gmail.com (Devaraj Manoj)

**Experimental**

**Synthesis of K_3_[Fe(CN)_6_]/CC**

To compare the electrochemical redox behaviour of CoFe-MOF/CC microelectrode, a control experiment was also carried in absence of cobalt chloride hexahydrate and trisodium citrate. In a typical reaction, K_3_[Fe(CN)_6_] (7.5 mM) was dissolved into 25 mL of MQ water to form a homogenous solution and followed by placing a precleaned activated carbon cloth. The reaction mixture was transferred into Teflon lined stainless steel autoclave and maintained at
80 ºC for 24 h to obtain K_3_[Fe(CN)_6_/CC electrode.


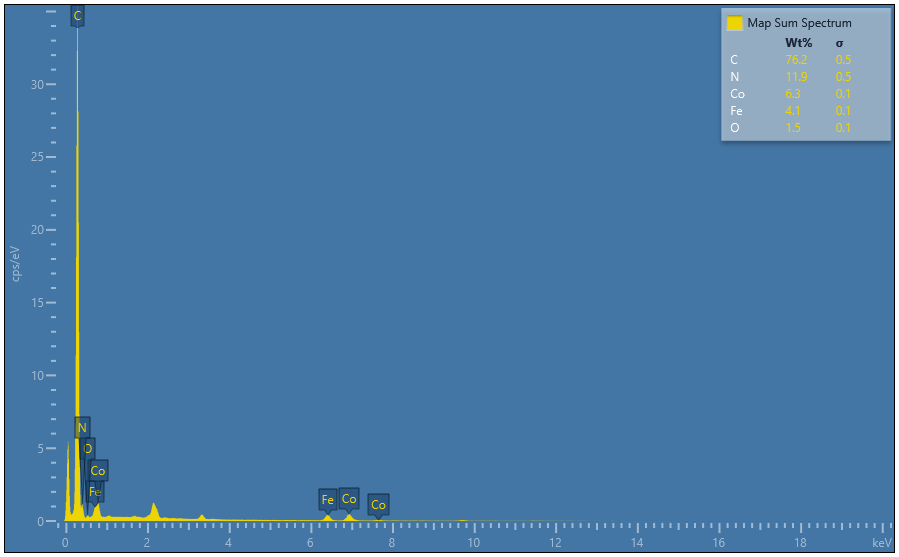


**Fig. S1.** EDX spectrum of CoFe-MOF/CC
